# Reducing Time to Diagnosis of Rare Genetic Diseases in a Medically Underserved Hispanic Population- Lessons Learned for Meaningful Engagement

**DOI:** 10.21203/rs.3.rs-3699740/v1

**Published:** 2023-12-13

**Authors:** Blake Vuocolo, Roberta Sierra, Dan Brooks, Christopher Holder, Lauren Urbanski, Keila Rodriguez, Jose David Gamez, Surya Narayan Mulukutla, Lori Berry, Ana Hernandez, Alberto Allegre, Humberto Hidalgo, Sarah Rodriguez, Sandy Magallan, Jeremy Gibson, Juan Carlos Bernini, Melanie Watson, Robert Nelson, Lizbeth Mellin-Sanchez, Hongzheng Dai, Claudia Soler-Alfonso, Kent Carter, Brendan Lee, Seema R. Lalani

**Affiliations:** Baylor College of Medicine; Baylor College of Medicine; Baylor College of Medicine; Baylor College of Medicine; Baylor College of Medicine; The University of Texas Rio Grande Valley; DHR Health; DHR Health; The University of Texas Rio Grande Valley; The University of Texas Rio Grande Valley; The University of Texas Rio Grande Valley; The University of Texas Rio Grande Valley; The University of Texas Rio Grande Valley; The University of Texas Rio Grande Valley; The University of Texas Rio Grande Valley; Texas Children’s Hospital; Milestones Therapeutic Associates; The University of Texas Rio Grande Valley; University of Florida Department of Pediatrics; Baylor College of Medicine; Baylor College of Medicine; The University of Texas Rio Grande Valley; Baylor College of Medicine; Baylor College of Medicine

**Keywords:** Virtual access to genomics, Consultagene, genome sequencing, medically underserved population

## Abstract

**Background:**

The utilization of genomic information to improve health outcomes is progressively becoming more common in clinical practice. Nonetheless, disparities persist in accessing genetic services among ethnic minorities, individuals with low socioeconomic status, and other vulnerable populations. The Rio Grande Valley at the Texas-Mexico border is predominantly Hispanic with a high poverty rate and an increased prevalence of birth defects, with very limited access to genetics services. The cost of a diagnosis is often times out of reach for these underserved families. Funded by the National Center for Advancing Translational Sciences (NCATS), Project GIVE (**G**enetic **I**nclusion by **V**irtual **E**valuation) was launched in 2022 to shorten the time to diagnosis and alleviate healthcare inequities in this region, with the goal of improving pediatric health outcomes.

**Methods:**

Utilizing Consultagene, an innovative electronic health record (EHR) agnostic virtual telehealth and educational platform, we designed the study to recruit 100 children with rare diseases over a period of two years from this region, through peer-to-peer consultation and referral.

**Conclusions:**

Project GIVE study has allowed advanced genetic evaluation and delivery of genome sequencing through the virtual portal, effectively circumventing the recognized socioeconomic and other barriers within this population. This paper explores the successful community engagement process and implementation of an alternate genomics evaluation platform and testing approach, aiming to reduce the diagnostic journey for individuals with rare diseases residing in a medically underserved region.

## Background

Rare genetic diseases are known to disproportionately affect children, causing early childhood death or chronic physical and/or neurodevelopmental challenges. Diagnosing children in a timely manner is crucial for providing families the opportunities for disease-specific intervention, appropriate counseling about recurrence risks, and addressing the psychosocial and financial challenges that are known to be associated with diagnostic odysseys [1]. There is substantial evidence indicating that disparities in genetic testing have a notable impact on health outcomes in disadvantaged areas worldwide [2, 3]. Within the United States healthcare system, low-income communities and ethnic minorities often receive subpar genetic services [4, 5].

According to Texas Birth Defects Epidemiology & Surveillance, over 24,000 babies are born each year in Texas with birth defects. While cutting-edge diagnostic tools for personalized medicine are accessible to high income and insured families in the state, there currently exists a substantial gap in the access to genomic healthcare for the minority community in the South Texas region, which is predominantly Hispanic. This region encompasses 8.3% of the population of Texas and includes 28 counties with a population of over 2.4 million people. Located along the southernmost tip of the Texas-Mexico border is the Rio Grande Valley (RGV), which has a population of about 1.4 million residents, and encompasses four counties (Starr, Cameron, Hidalgo, Willacy) with high concentrations of colonias, defined as residential areas that lack basic living necessities such as potable water and sanitation infrastructure. Over 94% of the population in the RGV is Hispanic, 34% of individuals under the age of 65 years are uninsured, and upwards of 25% people live in poverty. According to the Texas birth defects registry (TBDR), the prevalence of birth defects in the RGV reported between 1999–2014 was around 474 cases per 10,000 live births, much higher than other counties in Texas. Multiple studies have shown that Hispanic and Latino individuals living in the United States are less likely to get genetic testing compared to non-Hispanic/Latino individuals [6–8]. There is a critical need to provide genetic services to pediatric patients in this region, yet current healthcare infrastructure is lacking. All four counties in the RGV are designated medically underserved areas (MUA) by the Health Resources and Service Administration (HRSA), indicating an inadequate amount of primary care services to address the health needs of population of a region. Access to genomic care is not only restricted by low socioeconomic status of the residents and shortage of primary care providers in this region, but also by limited familiarity of the frontline healthcare professionals with genetic disorders. Previous studies have indicated that primary care providers in a Federally Qualified Health Center generally found clinical advantages in providing genetic testing to their patients. However, a significant number of them lacked training in genetics and did not integrate genetic service into their practices [9]. Even when children with rare diseases are eventually identified for assessment, the reduced availability of highly trained and qualified board-certified geneticists in this region hampers patients’ access to crucial services and prompt diagnoses.

The interwoven socioeconomic and healthcare system barriers in this region often cause prolonged diagnostic odysseys, potentially resulting in preventable health declines that could have been addressed through available treatments or personalized medicine. This scenario also leads to missed opportunities for participation in clinical trials and recurrence of frequently devastating diseases within families. There is an urgent need to implement alternate modes of delivery of care that could be readily integrated into the workflow or frontline healthcare providers to improve the lives of children with genetic disorders.

The University of Texas Río Grande Valley (UTRGV) was established in 2013 to reduce the gap in healthcare for people in the RGV by providing clinical care and preventive medical services to rural and underserved areas. The school of medicine graduated its first class of physicians in 2020 [10]. In February of 2022, funded by the NIH, our research team at Baylor College of Medicine (BCM) partnered with UTRGV to transform the current clinical practice in the region by leveraging an academically developed virtual genetics service platform, named Consultagene. We designed Project GIVE (**G**enetic **I**nclusion by **V**irtual **E**valuation) to simplify patient pathways and provide state-of-the art genetic evaluation and genomic sequencing to reduce time to diagnosis (TTD) for children with rare diseases in this region, circumventing the known socioeconomic and healthcare system barriers. This paper explores the successful implementation of a virtual genomics evaluation platform and testing approach to alleviate the diagnostic journey for individuals with rare diseases living in a medically underserved region.

## Methods

### Implementing Consultagene in a Medically Underserved Area to Reduce TTD

The study is approved by the Institutional Review Board of Baylor College of Medicine (H-50430). Between 2016 and 2018, the study team at BCM designed Consultagene, an innovative electronic health record (EHR) agnostic virtual web-based mobile platform to improve access to genomic care and education to individuals with limited access to expert professionals [11]. This virtual platform offers a comprehensive complement of services, including peer-to-peer consultation, interpretation of genetic test results, genetic counseling, and telehealth services. To enhance accessibility, Consultagene integrates educational components through a variety of videos presented in multiple languages, including Spanish. This approach has ensured cultural and linguistic sensitivity, making the platform suitable for both clinical and research applications. Furthermore, the platform is designed to adhere to the Health Insurance Portability and Accountability Act (HIPAA) compliance standards. Since 2019, Consultagene has successfully engaged diverse patient populations requiring counseling for prenatal testing, preconception and *in vitro* fertilization considerations, as well as specialized consultations related to cancer genetics and neurodegenerative diseases such as Huntington’s and Alzheimer’s disease. The seamless integration of virtual care delivery, education, and capability of transfer of recorded health information represented a logical extension of our efforts in implementing this tool for pediatric genetics care in the RGV. The application of this virtual platform follows a provider-setting model, via peer-to-peer consultation, effectively bypassing some of the recognized barriers within this population.

Project GIVE was structured to recruit approximately 100 subjects over a 2-year timeframe from this medically underserved region ClinicalTrials.gov ID NCT05318222). Unlike traditional genetics referral processes, the referrer base for this study has been extended by our team to a wider network of healthcare professionals in this region, including not just physicians and nurse practitioners, but also medical assistants, early childhood interventionists (ECI), as well as physical, occupational, and speech therapists serving children for their complex long-term care in rehabilitation centers. After accessing the portal, the referrers are prompted to upload pertinent clinical information including available electronic health records of patients to assist the study team in ascertaining those most likely to benefit from genetic evaluation. The Project GIVE clinical team, consisting of geneticists and a genetic counselor at BCM, study coordinator at the RGV, and UTRGV pediatricians, convenes remotely on a weekly basis to review the referrals and ascertain those most likely to benefit from genetic evaluation. In the preceding two years, the study team has formulated an approach to accommodate the majority of referrals despite the challenges of receiving limited medical records from the offices and constraints of exceptionally busy primary care providers in the region.

### Recruitment through the Texas Birth Defects Registry (TBDR)

We have additionally established a collaborative partnership with the Texas Birth Defects Registry (TBDR), with the aim of identifying pediatric patients in the RGV diagnosed between 2015 and 2020 through this registry. The Texas Department of State Health Services’ TBDR plays a pivotal role in the public health surveillance system, particularly in identifying infants and pregnancies with birth defects within one year after delivery [12]. Covering approximately 380,000 births annually, the registry uses an active surveillance system, conducting routine visits to all delivery and pediatric hospitals, birthing centers, and midwife facilities throughout Texas to ascertain cases. Systematic reviews of medical records are conducted employing both the International Classification of Diseases (ICD) codes for birth defects and text descriptions pertinent to such conditions. Notably, approximately 60% of selected records undergo further review by board-certified clinical geneticists. The TBDR staff provides this information through a secure web portal to the study team, as approved by the Institutional Review Board of the Texas Department of State Health Services. Utilizing our established patient engagement process, our team reaches out to these families and facilitates their referral to Consultagene portal through collaboration with their pediatricians for comprehensive evaluation.

### Inclusion and Exclusion criteria

The study is open to pediatric patients residing in the RGV who are aged 18 or younger, primarily prefer English or Spanish, and who have a suspected underlying genetic cause for their complex medical presentation. Children who have been accepted into the study predominantly have neurodevelopmental disorders, often with other organ systems affected. Participation eligibility excludes individuals who are over the age of 18, those with a confirmed genetic diagnosis, children who are less likely to have Mendelian disorder, and those living outside of the Rio Grande Valley. Project GIVE has principally centered on the assessment of pediatric patients within an outpatient setting for this phase of the study. Neonates identified with suspected genetic disorders are directed to Consultagene for evaluation following their discharge from the hospital. Over the course of the study, the project team has engaged with referrers across various platforms to provide educational outreach, focusing on specific indications for Consultagene referrals.

### Delivering Virtual Genetic Evaluation with Genome Sequencing (GS) and Longitudinal Follow-up in an Underserved Community

**Figure 1:**
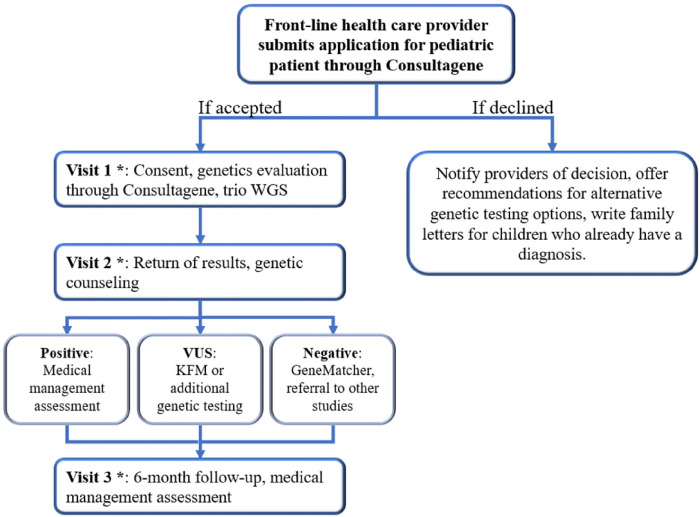
Project GIVE Evaluation Process

Upon acceptance into the study, participating families undergo a structured three-visit protocol over the span of one year. Figure 1 illustrates participant’s journey from referral to the final study visit. At the initial encounter, “Visit 1”, the pediatric participant and their family meet with the bilingual, bicultural research coordinator (R.S.) at the study site, UTRGV Pediatric Specialty Clinic in Edinburg, Texas, for the informed consent process. Families access the integrated educational videos in Consultagene platform in their preferred language. Two short videos are seen by all participants; (1) *Basics of Genetics*, (2) and *What to Expect at a Genetics clinic Visit*.

Following this, the clinical geneticists and genetic counselor, located in Houston, conduct an in-depth remote evaluation of the pediatric participant through the videoconferencing platform. Subsequently, buccal samples are collected for proband, duo, or trio genome sequencing (GS) and sent to Baylor Genetics, a Clinical Laboratory Improvement Amendments (CLIA)-certified laboratory in Houston, where a clinical report is generated. GS is performed with a mean depth coverage of 44x. Probands and biological family members may opt-in to receiving secondary findings in the 73 genes recommended by the ACMG SF v3.0 [13].

Upon receipt of the results by the clinical team in ~ 8 weeks, a videoconference return of results (ROR) call is scheduled with the families for “Visit 2”. Positive cases undergo an in-depth review of diagnosis and medical management, with accompanying informational letters crafted for both providers and patients in their preferred language. The Project GIVE clinical team facilitates follow up appointment referrals in the RGV whenever possible. In cases where it is clinically necessary, patients are directed to specialty clinics at Texas Children’s Hospital (TCH) in Houston, which is approximately 350 miles away from McAllen, TX. In instances where a variant of uncertain significance is ascertained, familial testing is undertaken to interpret the genomic results. In the event of a negative whole genome sequencing (WGS) result, a reanalysis of the data ensues, or the patient may be referred to additional research studies such as the Undiagnosed Diseases Network (UDN).

“Visit 3”, occurring 6 months post-disclosure, serves as a follow-up to collect additional medical information about the child and revisit the WGS results. Throughout this process, data through Clinical Sequencing Evidence-Generating Research (CSER) baseline, ROR, and follow up surveys are gathered to assess patient outcomes and impact of the study. A detailed summary of each visit is meticulously documented and promptly electronically faxed to the referring providers to facilitate effective communication.

### Enhancing Genomic Competency of Frontline Healthcare Professionals

A critical component of Project GIVE involves training and educating referrers to ensure expeditious patient referrals to Consultagene and is measurable with a quantifiable increase in referrals from primary care settings. In 2023, we developed and delivered two Continuing Professional Education (CPE) seminars designed to cater to the unique needs of non-genetics providers in this region. The content centers around the importance of genetic testing and recognition of rare diseases in pediatric patients, emphasizing genomic aspects relevant to clinical practice. Additionally, we introduce participants to machine-assisted technology such as Face2Gene to identify suitable patients for genetics referrals. Attendees also receive updates on the regional impact of Project GIVE. Leveraging established community networks of UTRGV and other partnerships, we have been able to disseminate information about the educational activity effectively. For ease of access, both CPE seminars, including Ethics credit, have been delivered at a public library auditorium in McAllen in the region. We also actively engage attendees in meetings and seek their input in selecting future curricula, fostering a collaborative and impactful approach. Our goal with these events is to assist the frontline healthcare professionals develop greater confidence in referring patients to genetics, understanding different genetic testing methodologies, and interpreting results to answer families’ questions (Fig. S1).

### Expected Study Outcomes

The primary outcome measure of Project GIVE is TDD of rare diseases, with the starting point designated as the date when a provider initially refers a patient to Consultagene. Patients will be longitudinally followed for a duration of up to one year post-referral. Event time will be computed in months as the time from referral to the date of ROR. We will assess patients’ and providers’ experiences after receiving genomic results using harmonized measures developed by the CSER consortium and designed to study the effectiveness of integrating GS into the clinical care of ethnically diverse and medically underserved individuals [14–16]. These measures are known for their cultural sensitivity, suitability for participants with low literacy levels, and are fully translated into the Spanish language. Analyses of parental measures will assess patient experience and satisfaction following return of results. Survey data will be collected to characterize sociodemographic variables, literacy, understanding, perceived utility of sequencing, and satisfaction with the ROR process.

## RESULTS

Over the course of 22 months, between Feb 2022 and November 2023, 169 pediatric patients (ages 0–18 years) with suspected undiagnosed rare diseases in the RGV have been referred through the Consultagene portal (Consultagene.org) for peer-to-peer consultation. Of these, 133 families have been accepted (78%); 83 families have been consented into the study, and 51 families have completed “Visit 1.” Among these, 98% of families identify themselves as Hispanic, and for 40% of participants, Spanish is the preferred language for communication with healthcare providers. About 51% of enrolled families report annual household income in the last year as <$20,000. Approximately 80% of accepted patients predominantly present with a neurodevelopmental (NDD) phenotype, including intellectual disability, seizures, developmental delay, autism spectrum disorder, behavioral/mental health concerns, and neuromuscular disease. WGS results have been received for 44/51 (86%) families. Overall, 13 families have received a confirmed diagnosis and 1 additional patient is categorized as partially solved. The diagnostic yield currently is ~ 32% (14/44), with average TTD of 192 days. Autosomal recessive disorders accounted for 4/14 (28%) of all confirmed diagnoses, including QARS1-related progressive microcephaly with seizures and cerebral and cerebellar atrophy (MIM# 615760), spastic tetraplegia, thin corpus callosum, and progressive microcephaly related to *SLC1A4* ( MIM# 616657), Seckel syndrome 8 (MIM# 615807), and vitamin D-dependent rickets, type I (MIM# 609506) due to *CYP27B1* variants. Two individuals with microdeletion syndrome have been ascertained in our study (14%); 46,XY sex reversal 1 due to SRY deletion (MIM# 400044) and Angelman syndrome (MIM# 105830). Other notable disorders include *TUBB3*-related cortical dysplasia complex, with other brain malformations 1 (MIM# 602661), *USP9X*-related X-linked intellectual developmental disorder-99 (MIM# 300919), Koolen-De Vries syndrome (MIM# 610443), and hypochondroplasia (MIM# 146000). All diagnosed patients have NDD phenotype with or without other medical concerns. At least, five additional cases appear possibly solved, awaiting known familial variant testing and other confirmatory studies currently. The remaining unsolved cases include three potentially novel disease genes, pending further characterization in larger gene-specific cohorts, and 1 medically actionable finding. Brugada syndrome (MIM# 601144) with a likely pathogenic *de novo* SCN5A variant was incidentally identified in a 2-year old female with global developmental delay. Within the limited cohort of families who received results and completed surveys for ROR (n = 26), a predominant number of families reported experiencing relief upon receiving genetic test results, emphasizing comprehension of the information provided. Furthermore, they expressed assurance in the doctors’ ability to apply these results for the improvement of children’s health. Here are some notable quotes from parents: [215] *Project GIVE has given us the hope we lost for over 12 months…. Since finding out our results, we have started treatment and created a health plan to promote the growth and development he lost. I could not be more grateful to all the research counselors, doctors and social workers who helped organize this experience.”*

[062]“Project GIVE gave my family and I not only hope but peace of mind. We were able to finally receive a diagnosis after years of trying to find an answer. If it weren’t for Project GIVE, I don’t think we would be able to assist our son in getting the head start he needs to thrive and grow. I can’t put into words how thankful we all are.”

[17] “I am very grateful for Project GIVE. I was given a diagnosis and information that no one could give to me.”

## Discussion

**Figure 2:**
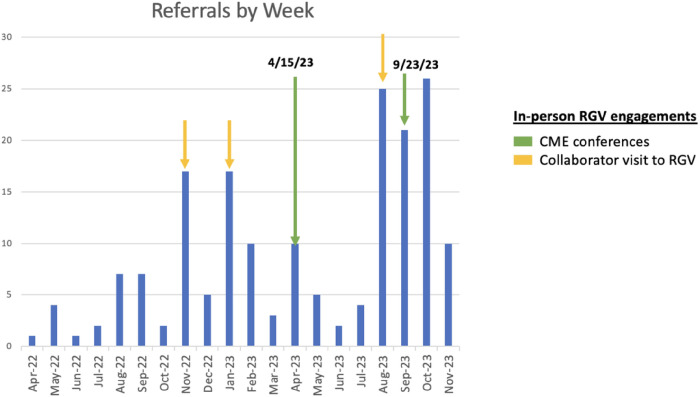
Progressive increase in patient enrollment since initiation of the study.

The integration of genomic information to enhance health outcomes is becoming more prevalent in clinical practice across the country. Nevertheless, the disparity in accessing genetic services among ethnic minorities and individuals with low socioeconomic status has resulted in the marginalization of the most vulnerable populations. Consultagene has emerged as a transformative solution, positioned to contribute significantly to reducing TTD in underserved communities with limited resources. Given the scarcity of local access to clinical geneticists in the region, this advanced referral tool has played a pivotal role in streamlining patient pathways and enabling cutting-edge evaluation of pediatric rare diseases within a provider-setting model. It represents one of the first systematic initiatives to integrate virtual health delivery with GS, particularly focused on a medically underserved pediatric population affected by rare diseases. Our efforts in extension of the referrer base to a wider network of healthcare professionals, which includes physicians, nurse practitioners, medical assistants, and early childhood interventionists (ECI) in this region, has significantly expedited the referral of patients requiring urgent evaluation. This strategic inclusion has enhanced the accessibility and efficiency of our services, ensuring timely referral for those in immediate need of assessment. Through the engagement of a diverse group of referrers such as ECI therapists providing physical, occupational, and speech therapies to children aged 0–3 years, we have clearly surpassed our recruitment targets. Consequently, we have improved the prospect of reducing TTD, particularly among the younger children who are most likely to benefit from genomic diagnoses. Ongoing in-person interactions with the community, including educational lectures and regular CPE conferences, have cultivated strong connections with healthcare providers in the region, leading to an incremental increase in Consultagene referrals over time (Fig. 2).

A recognized challenge in initiating a genomic study in an underrepresented region involves establishing trust among medical providers and community members [17]. The UTRGV clinicians, who are already trusted medical professionals in the community, have continually utilized their networks in the region to help ascertain children most likely to most benefit from Project GIVE. We have fostered robust relationships with community partners in the region through multiple in-person meetings with UTRGV and community pediatricians and subspecialists including endocrinologists, cardiologists, developmental pediatricians, pulmonologists, otolaryngologists, and audiologists. Leveraging the cultural significance of food in Hispanic traditions, we have incorporated this aspect into our workflow to enhance relationship-building. We have also formed partnerships on terms agreeable to mutual parties, including the development of service agreements as necessary. Furthermore, we have included our partners as co-authors on research disseminations for Project GIVE.

Within our research team, we are fortunate to have a bilingual, bicultural study coordinator native to the RGV, having an intimate understanding of the region’s culture and challenges. She has emerged as a trusted member of our group, fostering strong connections with both providers and participants, especially those who prefer communication in Spanish.

We have made continued efforts in providing quality care for all patients who have been referred to us, regardless of their acceptance into the study. For individuals referred who have a previously identified diagnosis, we provide both provider and family letters that explain the diagnosis, inheritance pattern, and follow-up care. For patients who would benefit from alternative genetic testing options, we work with providers to facilitate the ordering of the most suitable test. Consequently, all patients referred to us will undergo an appropriate genetics workup.

### Recognized Challenges in the Operation of Project GIVE

Our team has identified specific barriers in the operation of a virtual genetics program in an under resourced region. There are challenges related to accessing medical records for participants, who often receive care from multiple specialists across different hospital systems.

Phenotyping is frequently limited during the referral process due to this constraint and reduced availability of comprehensive clinical notes from providers who operate busy clinics. Supporting ancillary investigations such as brain MRI, EEG records, X-rays, echocardiogram and genetic tests if done, are often unavailable to our team for the majority of the referred patients. In the absence of essential diagnostic records and given the potential impact on interpretation of WGS results, conducting a thorough remote clinical evaluation during Visit 1 remains crucial for a comprehensive assessment of the referred patient.

Despite building robust collaborations in the region, patient recruitment has experienced occasional downturns during the course of the study. This is attributed to the considerable patient workload of many healthcare practitioners in the region, leaving them with limited time to refer patients to Project GIVE. To address this, we have progressively simplified the process of referral through Consultagene, facilitating a more straightforward and less burdensome process for the referrers.

## Conclusions

In summary, Project GIVE has successfully increased access to care and shortened the TTD for children with rare diseases in a medically underserved, predominantly Hispanic/Latino population. We strongly believe that these initiatives are readily applicable and replicable in other regions with limited healthcare resources.

### Future directions

In the months ahead, our ongoing efforts will center on continued recruitment of pediatric patients with complex phenotypes. Concurrently, we are committed to analyzing the CSER survey data to gain a deeper understanding of parental experiences and perceptions regarding genetic testing. Furthermore, we are in the process of concluding a qualitative study that explores the obstacles and facilitators in accessing comprehensive medical care and social/educational services for these children. The combination of quantitative and qualitative data from our cohort will offer invaluable insights into the experiences of families in the RGV with children facing complex, undiagnosed diseases. This information is crucial for informing best practices to improve genomic health equity along the Texas-Mexico border.

## Abbreviations

Project GIVE: Genetic Inclusion by Virtual Evaluation
TTD: Time to Diagnosis
CPE: Continuous Professional Education
ECI: Early Childhood Intervention
RGV: Rio Grande Valley
CSER: Clinical Sequencing Evidence-Generating Research
UTRGV: University of Texas Rio Grande Valley
TBDR: Texas Birth Defects Registry
NDD: Neurodevelopmental delay
WGS: Whole Genome sequencing
